# Ethanolic Extract of Propolis Modulates Autophagy-Related microRNAs in Osteoarthritic Chondrocytes

**DOI:** 10.3390/ijms241914767

**Published:** 2023-09-30

**Authors:** Consuelo Arias, Luis A. Salazar

**Affiliations:** 1 Escuela de Kinesiología, Facultad de Odontología y Ciencias de la Rehabilitación, Universidad San Sebastián, Santiago 8380000, Chile; consuelo.arias@gmail.com; 2 Center of Molecular Biology and Pharmacogenetics, Department of Basic Sciences, Faculty of Medicine, Universidad de La Frontera, Temuco 4811230, Chile

**Keywords:** osteoarthritic, autophagy, miRNA, propolis

## Abstract

Osteoarthritis is a multifactorial joint disease characterized by degeneration, and aging stands as a significant risk factor. Autophagy, a crucial cellular homeostasis mechanism, is influenced by aging and closely linked to cartilage health. This correlation between autophagy, cell death, and OA underscores its relevance in disease progression. MicroRNAs have emerged as autophagy regulators, with miRNA-based interventions showing promise in preclinical models. Remarkably, the ethanolic extract of propolis exhibits positive effects on autophagy-related proteins and healthy cartilage markers in an in vitro osteoarthritis model. The aim of this brief report was to evaluate through in silico analysis and postulate five microRNAs that could regulate autophagy proteins (AKT1, ATG5, and LC3) and assess whether the ethanolic extract of propolis could regulate the expression of these microRNAs. Among the examined miRNAs (miR-19a, miR-125b, miR-181a, miR-185, and miR-335), the ethanolic extract of propolis induced significant changes in four of them. Specifically, miR-125b responded to EEP by counteracting IL-1β-induced effects, while miR-181a, miR-185, and miR-335 exhibited distinct patterns of expression under EEP treatment. These findings unveil a potential link between miRNAs, EEP, and autophagy modulation in OA, offering promising therapeutic insights. Nevertheless, further validation and clinical translation are warranted to substantiate these promising observations.

## 1. Introduction

Osteoarthritis (OA) is the most common form of arthritis, significantly contributing to disability and a diminished quality of life [1,2]. According to the CDC (Centers for Disease Control and Prevention), the age-adjusted prevalence of arthritis in the USA in people older than 18 years is 23.7%, and of these, more than 30% are obese [1,2]. Although the common belief often attributes OA to cartilage deterioration, it actually affects the entire joint, leading to dysfunction [2].

OA is the final common pathway for traumatic and aging damage to the synovial joints [3]. Notably, aging is a prominent non-modifiable risk factor in OA development [2,3,4,5,6]. The aging process entails intricate physiological changes characterized by low-grade chronic systemic inflammation, culminating in the gradual loss of tissue structure and function. These changes create an environment conducive to conditions such as OA [3].

Cellular senescence, a hallmark of aging, emerges as a causal factor for various age-related diseases, including cancer, fibrosis, obesity, type 2 diabetes, and neuro-musculoskeletal conditions like OA [7]. In the context of cartilage, the progressive accumulation of cellular senescence with advancing age engenders disruptions in essential tissue processes such as autophagy [8]. This cascade of events leads to the characteristic structural and functional alterations observed in aging cartilage.

Autophagy, a critical mechanism responsible for maintaining cartilage homeostasis by eliminating dysfunctional organelles and macromolecules, is regulated by a complex network of proteins [9,10,11]. This highly dynamic process is essential for cell maintenance and is tightly controlled by various proteins [12]. However, the aging process leads to a decline in autophagic capacity within cells, resulting in altered cellular maintenance processes that trigger the generation of reactive oxygen species (ROS) and oxidative stress [13]. Both human and mouse aged cartilage exhibit diminished expressions of autophagic proteins [14].

In the context of OA, there is a pronounced reduction and loss of key autophagy-related proteins including ULK1, BECN1, and LC3, which correlates with an increase in chondrocyte apoptosis [13,15]. Furthermore, aged cartilage experiences a reduced expression of autophagy-related proteins such as ATG5 and LC3, leading to escalated apoptosis and structural deterioration. These alterations are believed to stem from disruptions in the autophagy pathway that accumulate in an age-dependent manner [16]. Intriguingly, research from our laboratory has demonstrated that chondrocytes subjected to IL1β stimulation exhibit an altered expression of three autophagy-associated proteins, LC3, ATG5, and AKT1, resulting in increased expression levels [17]. This aligns with other studies suggesting that heightened autophagy serves as an adaptive response, guarding cells against stress and regulating alterations in the expression of genes associated with OA [18]. Consequently, strategies aimed at restoring autophagic activity may hold promise in protecting against OA development [11].

Non-coding RNAs (ncRNAs) are RNA molecules that do not encode proteins. They are functional RNA molecules that play vital roles in cellular processes, including transcription and translation. The most important ncRNAs that have been identified in the regulation of gene expression in transcriptional and post-transcriptional levels include microRNAs (miRNAs), small interfering RNA (siRNA), piwi-interacting RNA (piRNA), long ncRNA (lncRNA), among others [19]. Notably, miRNAs have emerged as vital regulators of autophagy, with miRNA-based therapies showing promise in preclinical models [20]. These therapeutic strategies include reducing miRNA levels via anti-miRNAs or antagomirs oligonucleotides, as well as enhancing miRNA expression through miRNA mimics [21].

The involvement of miRNAs in autophagy regulation within chondrocytes, and how this regulation is perturbed in the context of OA, has been extensively elucidated. Numerous miRNAs have been identified as enhancers of the autophagy pathway, with altered expression in OA-afflicted chondrocytes. These miRNAs include miR-21 [22]; miR-27a [23]; miR-31-5p [24]; miR-140-5p [25,26]; miR-140-3p [27]; miR-146a-5p [28]; miR-149 [25]; miR-335-5p [29]; miR-766-3p [30]; and miR-411 [31]. Conversely, certain miRNAs have been implicated as inhibitors of this pathway, including miR-17-5p [32]; miR-20 [33]; miR-128a [34]; miR-155 [35]; miR-206 [36]; miR-375 [37]; and miR-378 [32]. The intricate mechanisms through which these miRNAs exert their effects are detailed in Table 1.

In the context of OA, where the available drugs have limitations and many clinical trial candidate drugs have failed to show efficacy or have adverse effects [15], researchers are exploring alternative approaches. One such approach is targeting epigenetic defects that occur early in various diseases, including OA [40]. Dietary phytochemicals, like polyphenols found in propolis, are being investigated for their potential to influence genetic and epigenetic processes [41]. Polyphenols are suggested as natural senolytics with low toxicity [7]. Their beneficial effects are attributed to antioxidant and anti-inflammatory properties and their ability to regulate the autophagy pathway [17,42,43,44]. Among compounds rich in polyphenols, propolis stands out for its diverse pharmacological properties, including hepatoprotective, antioxidant, and anti-inflammatory effects [45]. Propolis is a mixture of substances used by bees to defend the hive. The chemical composition of propolis is closely related to the resins and balsams of plant sources used to produce it [46]. It possesses a broad spectrum of pharmacological properties that make it potentially applicable to various pathologies [17,47,48].

Propolis could potentially regulate gene expression patterns [41,44]. Importantly, it is noted that Chilean propolis has been shown to modulate miRNAs [49], further reinforcing its potential to influence gene expression. Given these findings, since it has been described that propolis can regulate the autophagy pathway in chondrocytes with OA and that Chilean propolis performs some of its effects through miRNAs, we hypothesize that the ethanolic extract of propolis (EEP) could indeed regulate the autophagy pathway through miRNAs in chondrocytes with OA.

This study is the second part of an initial analysis already published on the effect of propolis on autophagy pathway proteins in chondrocytes, where changes in the expression of three proteins ATG5, AKT1, and LC3 were mentioned [17]. In the current study, we analyzed whether these effects could be mediated by changes in miRNAs. To elucidate this, an in-silico analysis was performed to identify potential miRNAs regulating autophagy-associated proteins, specifically ATG5, AKT1, and LC3, which are deregulated in the context of OA. Subsequently, we analyzed the effect of EEP on the expression of these miRNAs in IL1β-stimulated chondrocytes.

## 2. Results

### 2.1. Identification through In Silico Analysis of Candidate microRNAs

It had already been described that in cartilage, several proteins associated with the autophagy pathway are deregulated by aging [50]. Specifically, among the deregulated genes associated with aging in cartilage, three proteins—AKT1, ATG5, and LC3A—were selected due to their common use as markers for autophagy [17]. An in silico analysis was conducted using various bioinformatics tools to identify miRNAs that regulate these selected proteins. The analysis identified five miRNAs that regulate these genes in New Zealand rabbit (*Oryctolagus cuniculus*): miR-335, miR-19a, miR-181a, miR-125b, and miR-185 (Table 2).

### 2.2. EEP Treatment Changes the Expression of 4 of the 5 miRNAs Evaluated in Chondrocytes with OA

**Figure 1:**
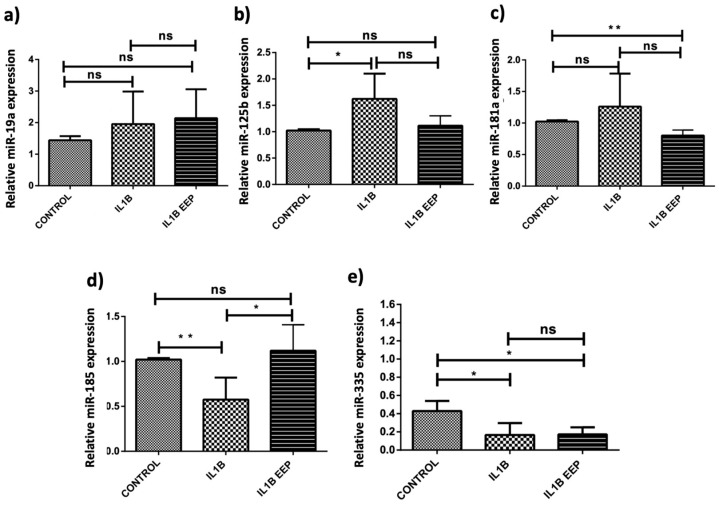
**Gene expression analysis of selected miRNAs in chondrocytes under different treatments.** The gene expression of specific microRNAs (miRNAs), including miR-19a (**a**), miR-125b (**b**), miR-181a (**c**), miR-185 (**d**), and miR-335 (**e**), was evaluated using real-time polymerase chain reaction (PCR). Chondrocytes were subjected to different treatments and analyzed after 24 h. The treatments included (1) control: no treatment; (2) IL1β-stimulated chondrocytes as an in vitro model of osteoarthritis (OA); and (3) IL1β-stimulated chondrocytes treated with an ethanolic extract of propolis (EEP). To determine the ratio of gene expression, the values of interest were referenced against basal conditions. The data are presented as the mean ± standard deviation (SD) of triplicate values. * *p* < 0.05; ** *p* < 0.005; N = 5 (independent experiments).

Five miRNAs—miR-335 (LC3), miR-19a and miR-181a (ATG5), and miR-125b and miR-185 (AKT1)—were analyzed in three conditions: control (untreated cells), IL1β-stimulated chondrocytes (representing an in vitro model of OA), and IL1β-stimulated chondrocytes treated with EEP. The results indicate that EEP treatment led to significant changes in the expression of four out of the five evaluated miRNAs. miR-19a did not show significant changes in any of the three conditions (Figure 1a). On the other hand, miR-125b expression significantly increased in IL1β-stimulated chondrocytes and was reduced to basal levels with EEP treatment (Figure 1b). miR-181a expression did not significantly change in IL1β-stimulated chondrocytes, but its expression significantly decreased with the addition of EEP treatment (Figure 1c). By contrast, miR-185 expression significantly decreased in IL1β-stimulated chondrocytes and increased to basal levels through EEP treatment (Figure 1d). The expression of miR-335 remained significantly decreased in IL1β-stimulated chondrocytes even with the addition of EEP treatment (Figure 1e).

## 3. Discussion

OA is a prevalent joint disorder characterized by the progressive degradation of articular cartilage, leading to pain, functional impairment, and reduced quality of life [30]. Recent studies have shed light on the intricate interplay between miRNAs, autophagy, and chondrocyte homeostasis, offering insights into potential therapeutic strategies [30,33,51].

Propolis is a resinous substance produced by bees, primarily the *Apis mellifera* species, through the collection and processing of various plant-derived materials [52]. The biological activity of propolis is intricately linked to its chemical composition, a composition profoundly influenced by the specific source plant or plants from which bees collect resin [53]. The beneficial effects of this compound have been previously reported. Clinical studies have shown beneficial effects of propolis oral treatment in relation to different respiratory diseases in patients [54,55,56] and to the alleviation of depression-related symptomatology [57], as well as beneficial effects on various metabolic diseases [58,59]. These findings strongly support its potential application in age-related diseases. EEP, as a complex mixture of various polyphenols, may owe its observed effects to the synergy between multiple compounds [17,60], those that can be recognized in other natural products such as pine needle cone extract [61,62].

The EEP used in this study was previously characterized [17]. High concentrations of pinocembrin, pinobanksin, and caffeic acid were reported. Some of these polyphenols have already been described as being able to regulate different targets of the autophagy pathway [63]; specifically, in the case of Chilean propolis, it is suggested that it is able to regulate this pathway [17], and in a study model of angiogenesis, it is suggested that it is able to regulate the expression of specific miRNAs [49].

This study initially focused on the search, through in silico analysis, for five miRNAs targeting autophagy-related proteins, including LC3, ATG5, and AKT1, which are known to be pivotal players in the autophagy pathway [51]. As a result, we identified miR-335, miR-19a, miR-181a, miR-125b, and miR-185 (Table 2). To decipher the response of the pre-selected miRNAs in an in vitro model of OA, the effects of IL-1β stimulation and EEP treatment in chondrocytes were explored. We were able to observe that the treatment with EEP changes the expression of 4 of the 5 miRNAs (Figure 1).

The miRNA-19 family has been implicated in diverse cellular processes, including autophagy and apoptosis [64]. However, in our study, miR-19a does not show significant changes in the conditions tested (Figure 1a). This suggests that miR-19a might not be directly affected by IL-1β or EEP treatment in the context of autophagy modulation.

The miR-125 family, known for its involvement in various diseases [65,66], also holds significance in the context of OA [65,67]. miR-125b, a member of this family, exhibits complex expression patterns in different cellular contexts. mir-125b is included in senescence-associated miRs (SA-miRs) and is associated with mitochondria (mitomiRs) [68]. Some studies in cancer models suggest miR-125b modulation of the autophagy pathway. It has been described that miR-125b could induce autophagy through its interaction with Foxp3 in thyroid cancer cells [69] and modulate autophagy in drug-resistant ovarian cancer cells using directly targeted MAP kinase interacting serine/threonine kinase 2 (MKNK2) [70]. In hepatocellular carcinoma cells, miR-125b regulates resistance to chemotherapy through autophagy-associated mechanisms [71]. It is proposed that miR-125 could attenuate rheumatoid arthritis (RA) and regulate the PI3K/Akt/mTOR signaling pathway [72]. In OA cartilage, miR-125b expression has been reported to increase [73], but in chondrocytes with OA, it is reduced [74,75]. In this study, miR-125b expression increases with IL-1β stimulation and decreases to basal levels with EEP treatment. This decrease aligns with other research using curcumin as a natural anti-inflammatory compound where it is stated that it causes an inhibition of the p53-BCL2 pathway [76].

Another miRNA of interest is miR-181a, which has been associated with aging, antioxidant defense, and the regulation of autophagy-related pathways [77]. Several articles suggest that ATG5 is a target of miR-181a [78,79,80]. It is postulated that miR-181a is part of the miRNAs related to aging and regulates mitochondrial function and autophagy [68]. miR-181a is also identified as knee-OA-related key miRNAs [81]. miR-181a is involved in antioxidant defense and its expression is increased in IL1β-stimulated chondrocytes, in OA cartilage, and in OA peripheral blood [82]. A differential expression of miR-181a has also been described in the different stages of maturation of chondrocytes with an upregulation in hypertrophic chondrocytes [3]. It is postulated that miR-181a regulates the balance of WNT and BMP pathways in cartilage [83]. miR-181a also regulates the expression of endogenous CCN1 and ACAN genes with a repressive role in cartilage metabolism via CCN1 as well as ECM construction via aggrecan [84]. Other studies suggest that the use of miR-181a-5p antisense oligonucleotide (ASO) injection could reduce the cartilage damage via promoting SIRT1 [85], attenuating OA in different experimental models and exhibiting cartilage-protective effects in vitro and ex vivo [86]. On the other hand, it has been described that miR-181a is a novel regulator of autophagy: miR-181a inhibits ATG5 expression in SGC7901/CDDP cancer cells [87], regulates ATG5-induced myocardial autophagy [88], and blocks starvation and rapamycin-induced autophagy in MCF-7 cells [79]; the inhibition of miR-181a expression could increase autophagic flow [80]; the downregulation of miR-181a suppresses LC3 and ATG5 protein expression in A549/DDP cells through suppression of the PTEN/PI3K/AKT/mTOR pathway [89]; and its overexpression recovers LC3 and ATG5 protein expression by promoting PTEN/PI3K/AKT/mTOR signaling in the adenocarcinoma cell line [89]. Inflama-miRs are a subset of inflammation-associated miRs, including miR-181a, which can modulate inflammatory molecules such as IL-1α [68].

The result of this study suggests a tendency to increase miR-181a expression in IL-1β-stimulated chondrocytes, but this increase is not significant. However, EEP treatment significantly decreases miR-181a expression compared to the control condition (Figure 1c). This aligns with other studies indicating that reducing miR-181a could improve the cartilage condition [82,83,84,85].

MiR-185 has been linked to the AKT signaling pathway and autophagy induction in various contexts. It has been reported that miR-185 targets IGF1 to activate the PI3K/AKT signaling pathway in an animal model of Parkinson’s disease [90] and induces autophagy via AKT signaling in hepatocellular carcinoma [91]. On the other hand, it is suggested that miR-185 promotes myocardial fibrosis and that targeting miR-185-5p in mice abolished pressure-overload-induced cardiac interstitial fibrosis [92]. On the other hand, it is suggested that miR-185 is involved in oral cancer progression since its anti-miR could inhibit tumor progression [93]. In the case of intervertebral degeneration, it has been reported that the inhibition of miR-185 increases the expression of pro-autophagy factors (LC3 and Beclin-1) and pro-apoptosis factors (caspase-3 and Bax) [94]. In this study, miR-185 expression significantly decreased in IL1β-stimulated chondrocytes and increased to basal levels when EEP treatment was added (Figure 1d). This dynamic expression pattern underscores the intricate regulation of autophagy-related processes by miRNAs.

MiR-335 is known to regulate chondrogenic differentiation and autophagy [28]. miR-335 could regulate chondrogenic differentiation and it is suggested that it mediates the development of OA by targeting the HBP1 gene [95]. Another study suggests that miR-335 has a protective role on cartilage because it is able to inhibit OA-associated inflammation through activation of autophagy [28]. In this study, the expression of miR-335 is significantly decreased in IL1β-stimulated chondrocytes and remains so even when EEP treatment is added (Figure 1e). This reduced expression suggests that miR-335 is negatively influenced by inflammation.

While the presented study highlights the potential roles of specific miRNAs in the autophagy pathway and their modulation by EEP, it is essential to acknowledge that miRNA–mRNA interactions can be tissue-specific [11], and to translate these results clinically, they need to be validated and confirmed in human in vitro models, such as chondrocyte cell lines, FFPE sections, and animal and human experiments. In this regard, changes in miRNA expression do not necessarily translate into observable phenotypic changes. For this reason, the results obtained suggest some kind of regulation, but further analysis is needed to confirm this type of modulation.

On the other hand, the bioavailability of natural products and the impact they may have on the joints is an important element in estimating or inferring the effects of these compounds in vivo. In this sense, it has been described that as the main risk factor for OA is ageing, treatments that reduce the chronic inflammation that ageing itself generates could be interesting therapeutic strategies that could generate beneficial effects in a systemic way. It is suggested that propolis could be a therapeutic candidate in diseases associated with ageing, especially for its antioxidant and anti-inflammatory capacity [96].

Based on the results, it is suggested that the observed effects of EEP on autophagy-pathway-associated proteins in chondrocytes with OA could potentially be mediated through changes in miRNA expression. These findings highlight the potential of miRNAs, specifically those regulated by EEP treatment, to play a role in influencing the autophagy pathway in chondrocytes affected by OA.

## 4. Materials and Methods

### 4.1. Primary Culture

Adult male New Zealand rabbits (*Oryctolagus cuniculus*) were provided by the Bioterio of the University of Sao Paulo (USP). The rabbits were initially anaesthetized using propofol and subsequently euthanized using potassium chlorate 60 mg/kg. Normal cartilage was removed from the following joints: scapulohumeral, humeral radius ulnar, coxofemoral, and femoro-tibio-patellar. The cartilage was washed 3 times in a PBS1x solution with 5% antibiotic. The extracted cartilage was digested in a solution of 2 mg/mL of Protease Type XIV. Bacteria from *Streptomyces griseus* (Sigma Aldrich, St Louis, MO, USA) was placed in PBSX1 for 1.5 h, and 1.5 mg/mL of collagenase B (Roche, Meylan, France) was placed in basic medium DMEM at 37 °C overnight. Chondrocytes were then centrifuged at 1200 rpm for 8 min, removing the supernatant and washing the cells with medium. Cells were quantified with trypan blue in a cell counter and 1 million cells were cultured per 90 mm plate. The chondrocytes were cultured in DMEM/F12 (1:1 with 15% FBS plus 1% antibiotic mixture of penicillin/streptomycin) at a density of 1 × 105 cells/mL and incubated in a humidified atmosphere of 5% CO_2_ at 37 °C. Culture medium was changed every two days and each passage was made when the confluence reached between 80 and 90%. We only used the second passage of cells in all experiments.

### 4.2. OA Model

For the induction of biological changes similar to those occurring in OA in joint cells, chondrocyte culture with IL1β was used [97]. Cells were cultured for 24 h under the following conditions: control (no treatment), IL1β (10 ηg/mL IL1β), and IL1β and EEP (10 ηg/mL of IL1β and 2.5 μg/mL of EEP).

### 4.3. Ethanolic Extract of PROPOLIS (EEP)

Raw brown propolis was obtained from a mountainous area near the town of Cunco, La Araucanía, Chile (latitude −38°58′40.46″, longitude −72°1′15.73″). Briefly, propolis was mixed with 80% ethanol in a 1:3 *w*/*v* ratio in an amber bottle and incubated at 60 °C for 30 min in constant motion. The mixture was then filtered on Whatman No. 1 filter paper to separate the ethanolic extract (EEP) from the crude propolis residue. The extract was left overnight at 4 °C and then centrifuged to promote the precipitation of waxes and a soluble residue. The EEP was then rotaevaporated, freeze-dried, and reconstituted at a 50% *w*/*v* ratio with alcohol. The extract was stored at −20 °C protected from light.

### 4.4. miRNA Analysis

The extraction of total RNA enriched with small RNAs was performed using the mirVana kit (Life Technologies, Carlsbad, CA, USA), following the manufacturer’s instructions. Reverse transcription was performed using the II miRCURY LNA Universal RT MicroRNA PCR Universal complementary kit (Exiqon, Vedbaek, Denmark) according to the manufacturer’s instructions. qPCR was performed using the miRCURY LNA Universal RT microRNA PCR system (Exiqon, Vedbaek, Denmark). All primers were designed by the manufacturer (HSA-MIR-125B-5P, HSA-MIR-19A-3P, HSA-MIR-185-5P, HSA-MIR-181A-5P, HSA-MIR-335-5P; Exiqon, Vedbaek, Denmark). The parameters set in the ABI 7500 fast thermocycler (Applied Biosystem, Waltham, MA, USA) were selected according to the manufacturer’s instructions.

### 4.5. Statistical Analysis

Outlier detection was performed using the Grubbs test. The distribution of the data was evaluated using the Shapiro–Wilk or Kolmogorov–Smirnov normality test. If it was not possible to evaluate this parameter, only non-parametric tests were performed. The hypotheses were tested using one-way ANOVA, followed by Bonferroni’s post test, when the data followed a Gaussian distribution. Otherwise, the Kruskal-Wallis test was applied, followed by Dunn’s post test. The significance level considered for the analyses was α = 0.05.

## Figures and Tables

**Table 1:** miRNAs suggested to regulate the autophagy pathway through different mechanisms in OA chondrocytes.

miRNAs	Effect on the Autophagy Process	Ref.
miR-17-5p	Inhibits autophagy pathway because downregulation of its expression causes suppression of p62 expression.	[32]
miR-20	Inhibits chondrocyte autophagy by targeting ATG10 via PI3K/AKT/mTOR signaling pathway.	[33]
miR-21	Promotes chondrocyte autophagy, stimulating the expression levels of ATG3, ATG5, ATG12, and LC3B.	[22]
miR-27a	Promotes autophagy through PI3K/AKT/mTOR signaling.	[23]
miR-31-5p	Promotes chondrocyte autophagy and suppresses mTORC1 activation in an ERK-dependent manner by inhibiting SOX4.	[24]
miR-128a	Represses chondrocyte autophagy through hindered ATG12 expression.	[34]
miR-140-5p	Promotes autophagy via downregulating FUT1.	[25]
	Promotes autophagy in chondrocytes through an upregulation of GBRAP, an autophagy marker.	[26]
miR-140-3p	Promotes autophagy.	[27]
miR-146a-5p	Promotes autophagy by modulating the levels of ATG5, p62, LC3-I, and LC3-II; by targeting NUMB; and through SDF-1/CXCR4-induced chondrocyte autophagy.	[28,37]
miR-146a	Promotes chondrocyte autophagy via depressing Bcl-2 expression when miR-146a is induced by hypoxia in chondrocytes.	[38]
miR-149	Promotes autophagy via downregulating FUT1.	[25]
miR-155	Decreases autophagy flux in chondrocytes by modulating expression of autophagy proteins such as Ulk1, FoxO3, ATG14, ATG5, ATG3, Gabarapl1, and Map1lc3.	[29]
	Suppresses autophagy, reducing the expression of Beclin 1 and LC3B, increasing p62 expression, and activating the PI3K/Akt/mTOR signaling pathway.	[39]
miR-206	Inhibits autophagy via activating the IGF-1-mediated PI3K/AKT-mTOR signaling pathway.	[36]
miR-335-5p	Activates autophagy by increasing autophagy-related factors such as Beclin-1, ATG5, and ATG7.	[28]
miR-766-3p	Facilitates autophagy in the chondrocytes through apoptosis-inducing factor mitochondria-associated 1.	[30]
miR-375	Suppresses autophagy by regulating the expression of Beclin-1, LC3II, and p62.	[37]
miR-378	Represses chondrocyte autophagy by targeting ATG2a and Sox6.	[32]
miR-411	Promotes chondrocyte autophagy by regulating expression of LC3, ULK-1, P62, and Beclin-1 by targeting HIF-1alpha in chondrocytes.	[31]

p62: ubiquitin-binding protein p62; ATG10: Autophagy-Related 10; PI3K: phosphoinositide 3-kinase; AKT: AKT serine/threonine kinase; mTOR: mammalian target of rapamycin; ATG3: Autophagy-Related 3; ATG5: Autophagy-Related 5; ATG12: Autophagy-Related 12; LC3B: microtubule-associated proteins 1A/1B light chain 3B; FUT1: fucosyltransferase 1 ; GBRAP: gamma-aminobutyric acid-receptor-associated protein; NUMB: NUMB endocytic adaptor protein; SDF-1: C-X-C motif chemokine ligand 12; CXCR4: C-X-C motif chemokine receptor 4; Bcl-2: BCL2 apoptosis regulator; ULK1: Unc-51-like autophagy activating kinase 1; FoxO3: forkhead box O3; ATG14: Autophagy-Related 14; Gabarapl1: GABA-type-A-receptor-associated-protein-like 1; Map1lc3: microtubule-associated protein 1 light chain 3 beta; Beclin 1: Beclin 1; IGF-1: insulin-like growth factor 1; ATG2: Autophagy-Related 2; Sox6: SRY-box transcription factor 6; HIF-1alpha: hypoxia-inducible factor 1 subunit alpha.

**Table 2:** miRNAs selected through in silico tools that regulate autophagy pathway proteins (ATG5, AKT1, and LC3).

miRNA	GEN	Tools
miR-125b-5p	AKT1	mirWalk 2.0
	AKT1	miRTarBase
miR-185-5p	AKT1	mirWalk 2.0
	AKT1	miRtarBase
miR-19a-3p	ATG5	mirWalk 2.0
	ATG5	DIANA TOOLS
miR-181a-5p	ATG5	mirWalk 2.0
	ATG5	miRtarBase
miR-335-5p	MAP1LC3A	DIANA TOOLS
	MAP1LC3A	mirWalk 2.0
	MAP1LC3A	miRTarBase

## Data Availability

Data are contained within the article. Raw data and materials are available from the authors upon reasonable request.
